# The legacy of the Child Health and Nutrition Research Initiative (CHNRI)

**DOI:** 10.7189/jogh-06-010101

**Published:** 2016-06

**Authors:** Robert E Black

**Affiliations:** Institute for International Programs, Johns Hopkins Bloomberg School of Public Health, Baltimore, MD, USA

## Abstract

Under the Global Forum for Health Research, the Child Health and Nutrition Research Initiative (CHNRI) began its operations in 1999 and became a Swiss foundation in 2006. The vision of CHNRI was to improve child health and nutrition of all children in low– and middle–income countries (LMIC) through research that informs health policy and practice. Specific objectives included expanding global knowledge on childhood disease burden and cost–effectiveness of interventions, promoting priority setting in research, ensuring inclusion of institutions and scientists in LMIC in setting priorities, promoting capacity development in LMIC and stimulating donors and countries to increase resources for research. CHNRI created a knowledge network, funded research through multiple rounds of a global competitive process and published research papers and policy briefs. A signature effort was to develop a systematic methodology for prioritizing health and nutrition research investments. The “CHNRI method” has been extensively applied to global health problems and is now the most commonly used method for prioritizing health research questions.

In the early 1990s there was growing recognition that low– and middle–income countries (LMIC) continued to have longstanding threats from infectious diseases, malnutrition and maternal and perinatal conditions, but were also increasingly facing non–communicable diseases and injuries. Research was considered essential to address these diverse problems, but given limited resources and capacity it was thought that priorities must be set. In 1994 the World Health Organization named an Ad Hoc Committee on Health Research Relating to Future Investment Options. The report [1] issued by this Committee provided cogent arguments for better aligning research priorities with the global disease burden and building capacity for research, especially in LMIC. The report proposed a five step process to inform research and development resource allocation: 1) How big is the health problem?; 2)Why does the disease burden persist?; 3) Is enough known about the problem now to consider possible interventions?; 4) How cost–effective will these interventions be?; and 5) how much is already being done about the problem? These questions were usually asked broadly about a disease such as malaria or problem area such as emerging microbial threats. Others built upon that for research topics within these broad areas, but methods were not proposed to more systematically prioritize specific research questions. The call in this report for a focus on operational research to make existing interventions more efficient and responsive to the needs of households was largely unheard, possibly in part because the report itself named as “best buys” the development of new drugs, vaccines, tests and other technologies, rather than studies of how to enable health systems to deliver existing services more effectively and equitably.

As a follow–up to the Investing in Health Research and Development Report, The Global Forum for Health Research began as an international foundation headquartered in Geneva, Switzerland in 1997. Its aim was to increase the amount of research on global health issues. In its advocacy it pointed to the “10/90 gap”, identifying that only 10% of the world’s health research spending is targeted at 90% of global health problems. The Forum continued to promote the five step process to advocate for research and held international meetings on research. As part of its mandate, the Forum facilitated the creation of more specific research initiatives, one of which was the Child Health and Nutrition Research Initiative (CNHRI). Begun under the Forum in 1999, CHNRI became a Swiss foundation in 2006. The vision of CHNRI was to improve child health and nutrition of all children in LMIC through research that informs health policy and practice. Specific objectives included expanding global knowledge on childhood disease burden and cost–effectiveness of interventions, promoting priority setting in research, ensuring inclusion of institutions and scientists in LMIC in setting priorities, promoting capacity development in LMIC and stimulating donors and countries to increase resources for research. With an international foundation Board and a Secretariat, based sequentially in Geneva, Dhaka and New Delhi, CHNRI played an active role in Global Forum annual conferences, created a knowledge network, funded research through multiple rounds of a global competitive process and published research papers and policy briefs.

A signature effort of CHNRI was to develop a systematic methodology for prioritizing health and nutrition research investments. This method included asking a wide selection of stakeholders and experts for specific research questions addressing a topic area. These questions were then curated and scored for priority using criteria such as the question’s answerability and the resulting intervention’s effectiveness, impact on disease, contribution to equity and deliverability. The “CHNRI method” has been extensively applied to global health problems and is now the most commonly used method for prioritizing health research questions [2,3].

In the 15 years that CHNRI operated before the foundation was dissolved in 2015, there have been substantial increases in child health and nutrition research and more reliance on sound evidence for policy and programs. The capacity for research in LMIC has improved; much more capacity building is needed, especially because research funding for global problems has improved. There has been much greater use of systematic and transparent methods involving multiple stakeholders in prioritizing and focusing research funding. The CHNRI method may be a lasting legacy of the foundation and the efforts of its Board, Secretariat and many contributors.
